# Scientific Mapping of Global Research on Health Equity by 2023: A Bibliometric Study

**DOI:** 10.1002/hsr2.70478

**Published:** 2025-02-19

**Authors:** Yaser Sarikhani, Arefeh Kalavani, Seyede Maryam Najibi

**Affiliations:** ^1^ Research Center for Social Determinants of Health Jahrom University of Medical Sciences Jahrom Iran; ^2^ Department of Persian Medicine School of Medicine, Research Center for Traditional Medicine and History of Medicine Shiraz University of Medical Sciences Shiraz Iran

**Keywords:** bibliometric analysis, bibliometrics, health equity, research

## Abstract

**Background and Aims:**

There has been a growing focus on health equity (HE) within health systems. Studies in HE have revealed significant domains that have been the focus of attention for academics and research institutions. This research aimed to provide an in‐depth perspective on the research efforts conducted around the world concerning HE.

**Methods:**

In this bibliometric study, we used co‐word analysis to map HE studies indexed in Scopus, Web of Science, ScienceDirect, and PubMed by the end of 2023. A comprehensive search was carried out in the databases, and the data were analyzed employing VOSviewer software. Along with analyzing publication trends, thematic clusters, and emerging topics in HE were identified.

**Results:**

The compound annual growth rate of the HE documents in PubMed, Scopus, ScienceDirect, and Web of Science were 0.157, 0.173, 0.453, and 0.317, respectively. Topic clusters of HE keywords in the period preceding the COVID‐19 pandemic were “Health care,” “Health economics,” “Race and ethnicity,” “Social determinants of health,” and “Age and gender.” The analyses related to the time following the onset of the COVID‐19 pandemic resulted in the identification of six topic clusters, namely “Health workforce,” “Risk factors,” “Maternal and child health,” “COVID‐19,” “Cancer,” and “Mental health.” Moreover, “Artificial intelligence,” “Racial disparity,” “Machine learning,” and “COVID‐19,” were four key emerging topics of HE pertinent to the post‐COVID‐19 period.

**Conclusion:**

In recent years, there has been a significant increase in research focused on HE. The focus of research in HE has shifted to an emphasis on various diseases and their risk factors. Emerging topics identified in this study represent significant areas of interest as novel research domains, particularly within low‐ and middle‐income countries.

## Summary

- A substantial increase in research on health equity has been observed over the last few years.
- The focus of research in health equity has shifted from the fundamental aspects of equity in health, such as social determinants of health, to an emphasis on the impact of various diseases and their risk factors.
- Artificial intelligence, racial inequality, and machine learning are three emerging subjects in health equity, representing significant research opportunities, particularly in low‐ and middle‐income countries.

## 1 Introduction

Throughout history, equity has emerged as a critical issue for human societies, resulting in the establishment of multiple definitions that seek to articulate its fundamental nature [1]. The CDC defines health equity (HE) as “the state in which everyone has a fair and just opportunity to attain their highest level of health.” This concept emphasizes that achieving HE requires addressing social determinants of health (SDH) and eliminating health disparities, particularly for those historically marginalized or disadvantaged due to various factors such as economic status, race, or geographic location [2]. In this regard, the World Health Organization (WHO) defines HE as the absence of unfair, preventable, or amendable health differences among population groups, characterized by social, economic, demographic, or geographic factors, or other factors contributing to inequality [3].

There has been a notable increase in the emphasis on HE within the macro‐level policies of healthcare systems, despite the discrepancies in how this concept is articulated and understood [4]. Additionally, the body of research on HE has experienced considerable growth over the previous 30 years [5], resulting in extensive studies focused on the requirements and outcomes associated with HE [6]. The investigations conducted on HE represent a vital component of this domain, as they highlight significant topics of inquiry pursued by scholars and research organizations [7]. Recently, bibliometrics has emerged as a captivating field of research, marked by various practical applications [8]. This field provides an information‐based perspective on subjects through the process of knowledge mapping and assists in pinpointing current areas of interest and emerging trends [9].

A variety of bibliometric studies can be implemented to analyze the patterns of publication, citation, and collaboration within a given field of study. Citation and Co‐citation analyses are the two most common methods applied to evaluate the impact of publications. These analyses involve a detailed examination of citations within a specific set of publications to understand their influence on later research activities [10]. Content analysis serves as another method that involves the detailed analysis of publication content to reveal patterns in research, such as the methodologies employed, the theoretical frameworks considered, and the research questions that have been addressed [11]. Co‐word analysis is among the most frequently used bibliometric can be applied to discern emerging patterns and novel research areas. This method is a crucial reference for both researchers and those involved in research policy, drawing attention to the principal elements of a research topic and specifying the domains that require targeted investigation [12].

While the significance of HE in the health sciences continues to grow, and identifying less‐explored research areas is essential, there has been a dearth of bibliometric research focused on this topic. Two previous studies have been carried out to examine the research on health disparities in connection with the COVID‐19 pandemic [13] and to elucidate the connections between cultural concepts and HE [14]. Hence, this study utilized a co‐word analysis method to create a comprehensive scientific map of worldwide research on HE. This map is designed to provide researchers and science policymakers with essential insights regarding trends and research domains.

## 2 Methods

In this bibliometric study, we applied a co‐word analysis approach to map HE studies indexed in four leading databases, including Scopus, Web of Science, ScienceDirect, and PubMed. Co‐word analysis is a type of content analysis first proposed in 1986 to reflect the dynamics of science. According to this method, a map is created using the strength of correlation between keywords. The procedure of co‐word analysis includes several key phases. The first phase is terminological extraction, which focuses on identifying relevant keywords from the analyzed texts. This is succeeded by the computation of clusters, where the frequency and patterns of co‐occurrences among these keywords are analyzed to form clusters that reflect related concepts. The final phase involves strategic diagram construction, which visualizes the relationships and associations between these clusters, thereby providing insights into the evolution of themes over time [15].

The current study included all HE scientific reports indexed in the mentioned databases up to the end of 2023. Considering the analysis of all retrieved articles, no sampling was conducted, and the study sample incorporated all indexed reports. In light of the effects of the COVID‐19 pandemic on HE studies, the databases were explored for two varying time intervals. The initial period extends until the end of 2019, coinciding with the onset of the pandemic. In contrast, the subsequent period covers the duration from the beginning of 2020 to the end of 2023. The bibliometric analyses were conducted independently for each of the two specified periods.

To ensure a comprehensive search, the first step was identifying keywords pertinent to the research subject, leveraging the MeSH and Emtree databases. Utilizing these two databases, we identified keywords pertinent to the concepts of “health equity” and “health inequity.” In the next step, we combined all the terms associated with the expected concepts utilizing the logical operator “OR.” The specified terms were searched in title, abstract, and keywords (TITLE–ABS–KEY). Consequently, we utilized the following search strategy in our study.

TITLE–ABS–KEY (“Health equity” OR “equity, health” OR “equity in health” OR “health inequity” OR “inequity, health” OR “health inequities”)

To achieve a detailed and thorough analysis, complete bibliographic records were extracted from the databases and saved in RIS format. To eliminate duplicate citations, all files retrieved from the databases, categorized by two distinct periods, were imported into Endnote X9 software (Clarivate Analytics, Philadelphia, PA, USA), and the resulting output was subsequently saved in RIS format.

Data were analyzed using VOSviewer version 1.6.20 (CWTS, Leiden University, October 2023). To achieve a more accurate evaluation of the correlation among retrieved keywords and to enhance the reliability of the results, a criterion was set that necessitates at least five repetitions of a keyword for its inclusion in the co‐word analysis. Accordingly, the analysis encompassed 796 items associated with the period preceding the COVID‐19 pandemic and 862 items related to the timeframe following the pandemic's outbreak.

The keywords from the retrieved studies were clustered according to their co‐occurrence, enabling the identification of the predominant research area in HE. Furthermore, emerging topics were identified based on the frequency of keyword occurrences throughout a defined period. Finally, the calculation of the compound annual growth rate (CAGR) for HE publications, differentiated by database, was performed using the following formula:

$$\mathrm{CAGR}\;\left(t_{0},t_{n}\right)={\left(\frac{v_{\left(t_{n}\right)}}{v_{\left(t_{0}\right)}}\right)}^{\frac{1}{t_{n}-t_{0}}}-1$$

where $V_{\left(t_{0}\right)}$ corresponds to the number of articles released in the first year, $V_{\left(t_{n}\right)}$ represents the number of articles published in the last year, and *t_n_* – *t*_0_ indicates the time duration in years [16]. CAGR expresses the average annual change, resembling the compound interest mechanism found in savings accounts. Although it has been predominantly used in finance, its application has become more widespread in bibliometric studies because it highlights annual progress rather than absolute numbers, augmenting trendline analysis and enabling more precise conclusions [17, 18].

## 3 Results

After aggregating the data from four distinct databases and excluding duplicates, a total of 24,867 citations was prepared for the final analysis. The first publications related to HE were released in 1975, and both Scopus and PubMed databases contain two indexed items from that year. The highest volume of publications was indexed in the Scopus database (*N*: 22,409). Within the Scopus database, articles are the most frequently indexed document type, representing 67.6% of the total. Other types include reviews at 14.0%, notes at 8.8%, and editorials at 7.5%. The evaluation of the growth rate of the HE documents index in PubMed, Scopus, ScienceDirect, and Web of Science revealed that the CAGR for these databases is 0.157, 0.173, 0.453, and 0.317, respectively. The information related to the data extracted from the databases is detailed in Table 1.

**Table 1:** Number of articles retrieved from different databases.

	Year (number of citations)		Up to 2019^a^	2021–2023^b^	Sum	CAGR^c^
PubMed	1975 (2)	2023 (2629)	5045	8757	13,802	0.157
Scopus	1975 (2)	2023 (5165)	7348	15,061	22,409	0.173
ScienceDirect	2006 (1)	2023 (837)	937	2179	3116	0.453
Web of Science	1994 (1)	2023 (3887)	5330	10,693	16,023	0.317
Total	—	—	18,660	36,690	55,350	—
Duplicate deletion	—	—	10,407	20,076	30,483	—
Final citation analyzed	—	—	8253	16,614	24,867	—

^a^ Before COVID‐19 pandemic.

^b^ Since COVID‐19 pandemic.

^c^ Compound annual growth rate.

The Scopus database indicates that the highest volume of published research on HE, totaling 2004 papers (9.2% indexed publications), is attributed to Harvard Medical School, the University of Toronto, and the University of California. Furthermore, data from the Scopus and Web of Science databases indicate that the United States leads in the volume of publications on HE. Canada, the United Kingdom, and Australia follow occupying the second, third, and fourth ranks, respectively. Table 2 shows the countries with the largest share of publications on HE.

**Table 2:** Contribution of different countries to health equity publications based on the citation databases.

Scopus database		Web of Science database	
Country	Number of documents	Country	Number of documents
United States	11,411	United States	10,115
Canada	2532	Canada	2135
United Kingdom	2048	Australia	1324
Australia	1593	England	1324
Brazil	610	Brazil	475
New Zealand	552	New Zealand	469
Switzerland	500	China	363
China	497	Switzerland	313
India	443	South Africa	301
South Africa	417	Sweden	300

According to the co‐occurrence network analysis, we determined topic clusters of HE research keywords across two discrete temporal intervals, specifically before and after the onset of the COVID‐19 pandemic. As presented in Table 3, the network of the period preceding the COVID‐19 pandemic encompasses five distinct clusters. Clusters are labeled and ranked by the frequency of their keywords, which are “Health care,” “Health economics,” “Race and ethnicity,” “Social determinants of health,” as well as “Age and gender,” in that sequence.

**Table 3:** Topic clusters of the research in the field of health equity.

Period	Cluster (color)	Cluster label	Important topics	Number of terms
Up to 2019^a^	1 (Red)	Health care	Health care policy, Health care access, Health care quality, Health care cost, Health care system, Health care planning, Patient care, Public health services, Health care organization, Health care need	225
	2 (Green)	Health economics	Poverty, Economic, Health insurance, Income, Financial management, Health expenditures, Insurance, Health care financing, Universal coverage, Insurance coverage	181
	3 (Blue)	Race and ethnicity	Ethnic groups, Hispanic, Minority groups, Race, Racism, Race difference, African Americans, Minority health, American Indians, Indigenous	174
	4 (Yellow)	Social determinants of health	Residence characteristics, Health behavior, Social justice, Socioeconomic factors, Poverty, Environment, Social class, Capacity building, Climate change, Housing	154
	5 (Purple)	Age and gender	Female, Male, Middle aged, Adolescents, Aged, Young adults, Sex factor, Age factor, Age distribution, Sex distribution	62
2020–2023^b^	1 (Red)	Health workforce	Health care personnel, Physician, Medical staff, Professional development, Medical education, Leadership, Nurse, Capacity building, Multidisciplinary team	274
	2 (Green)	Risk factors	Obesity, Risk assessment, Physical activity, Exercise, Body mass, Diet, Geography, Tobacco use, Race factors, Healthy lifestyle	179
	3 (Blue)	Maternal and child health	Pregnancy, Newborn, Child health, Prenatal care, Maternal mortality, Infant mortality, Abortion, Maternal care, Perinatal care, Child development	168
	4 (Yellow)	COVID‐19	Coronavirus disease 2019, Pandemic, Infection control, SARS‐CoV‐2, COVID‐19 vaccines, Vaccination, COVID‐19 testing, Coronavirus infections, Virus transmission, Vaccine hesitancy	134
	5 (Purple)	Cancer	Cancer screening, Neoplasms, Cancer incidence, Breast cancer, Survival rate, Cancer control, Cancer mortality, Cancer risk, Cancer survival, Cancer therapy	58
	6 (Light blue)	Mental health	Psychology, Depression, Drug dependence, Mental disease, Anxiety, Substance use, Violence, Mental health care, Social stigma, Suicide	49

^a^ Before COVID‐19 pandemic.

^b^ Since COVID‐19 pandemic.

**Figure 1:**
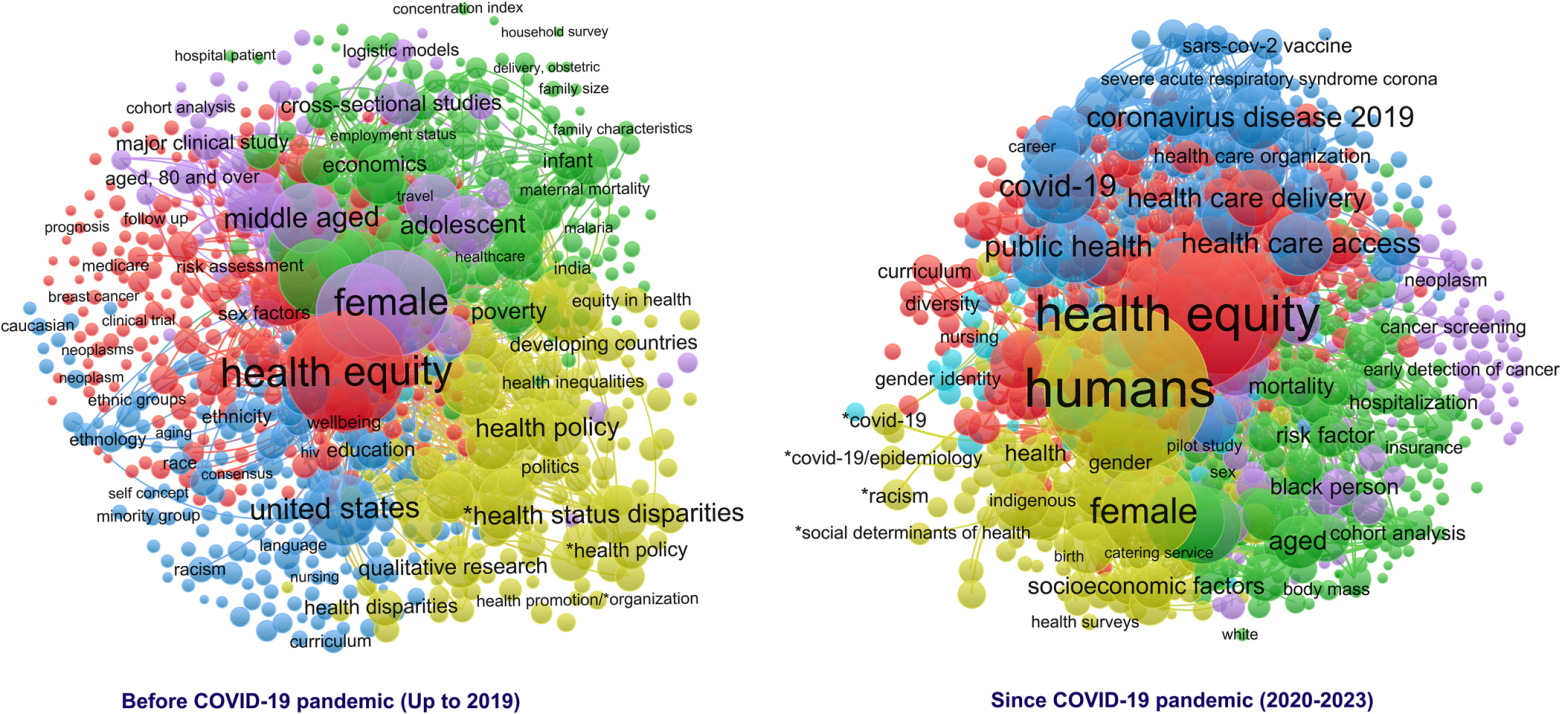
Co‐occurrence network of research keywords in the field of health equity.

The network associated with the period following the onset of the COVID‐19 pandemic comprises six distinct topic clusters. The identified clusters, arranged by the frequency of their associated keywords, include “Health workforce,” “Risk factors,” “Maternal and child health,” “COVID‐19,” “Cancer,” and “Mental health.” Figure 1 shows the co‐occurrence network of research keywords in HE.

**Figure 2:**
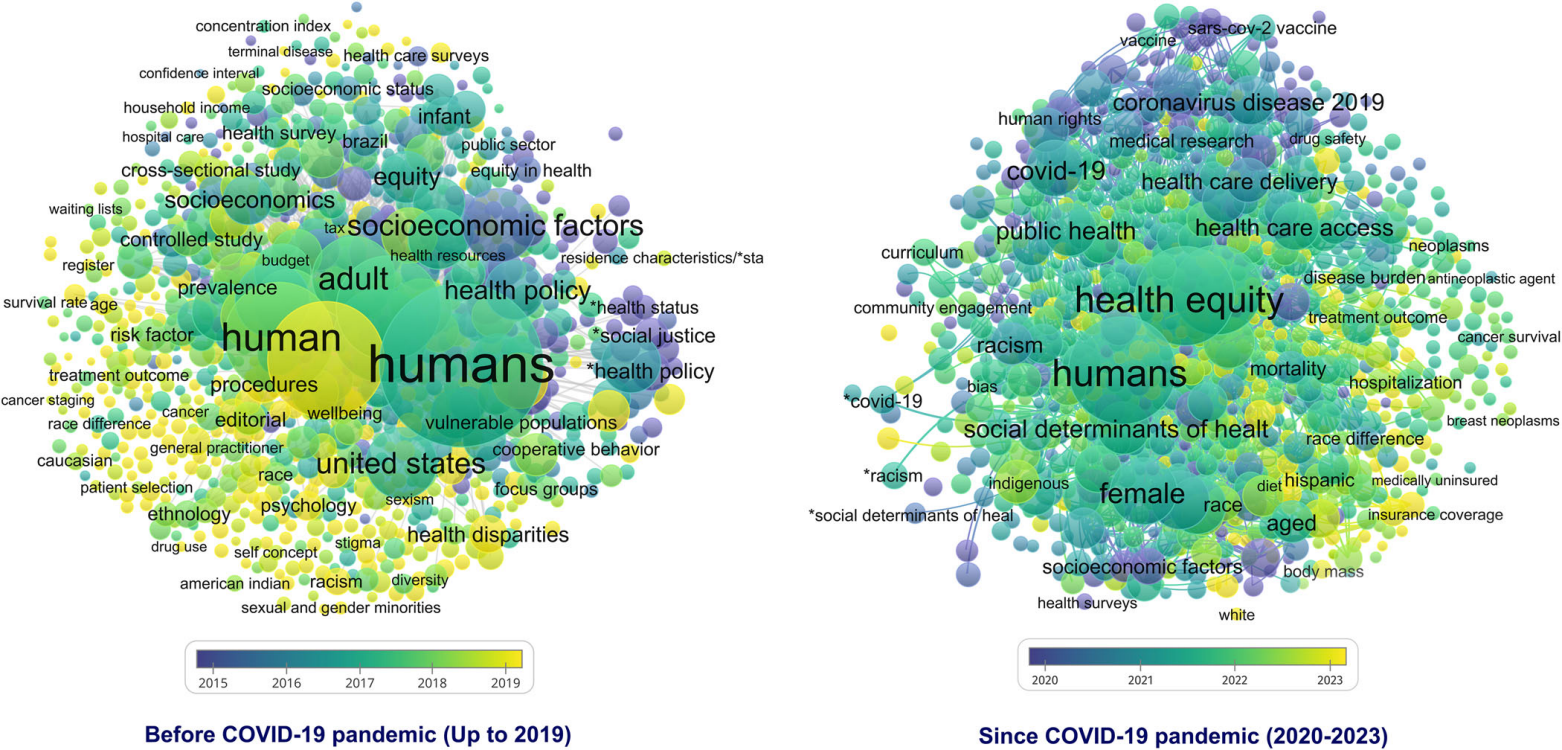
Emerging topics in the field of health equity.

We analyzed the emerging topics across the two distinct periods to identify which topics became more prominent over time. The data provided in Table 4 show that “Disease burden,” “Telemedicine,” “Sex factor,” “Sexual and gender minorities,” and “Stakeholder participation” were five emerging topics of research in HE before the COVID‐19 pandemic. Furthermore, “Artificial intelligence,” “Racial disparity,” “Machine learning,” “COVID‐19,” and “Electronic health record” were identified as five emerging topics pertinent to the period following the beginning of the COVID‐19 pandemic. Figure 2 shows the network of emerging topics in HE.

**Table 4:** Emerging topics in the field of health equity.

Before COVID‐19 pandemic (up to 2019)		Since COVID‐19 pandemic (2020–2023)	
Emerging topics	Frequency of terms (cluster number)	Emerging topics	Frequency of terms (cluster number)
Disease burden	36 (1)	Artificial intelligence	321 (1)
Telemedicine	34 (1)	Racial disparity	309 (2)
Sex factor	32 (5)	Machine learning	201 (1)
Sexual and gender minorities	26 (3)	COVID‐19	193 (3)
Stakeholder participation	26 (1)	Electronic health record	191 (1)
Early detection of cancer	24 (1)	Body mass	142 (2)
Palliative treatment	20 (1)	Electronic medical record	79 (1)
Global burden of disease	19 (1)	Economic inequality	75 (5)
Contagious disease	16 (2)	Information technology	71 (1)
Sexual and gender minority	15 (3)	Drug efficacy	68 (4)

## 4 Discussion

This research was carried out to analyze studies on HE that were indexed in four databases. In this regard, a bibliometric method was used to analyze the publication trend, identify topic clusters, and determine emerging topics. The study results reveal that the earliest research in HE was indexed in databases as early as 1975, with a significant rise in the number of publications observed over the past decade. Over the past 50 years, there has been a considerable upward trend in scientific research concerning HE, which has exceeded the average growth rate of general scientific output. This situation is likely connected to the growing interest among researchers in analyzing and comprehending the disparities in health over time, as well as the complexities inherent in this issue [19].

The investigation into the distribution of HE publications indicated that most of the research was produced by developed nations, with the United States and Canada occupying the top two positions. Researchers in the United States engage in the most significant collaboration with international counterparts, likely due to the substantial availability of research funding and the considerable number of researchers and scientists in the country [20]. However, the United States has persistently faced considerable and enduring racial inequalities in health coverage, mainly attributable to its socioeconomic and political circumstances [21, 22]. This fact may contribute to the understanding of the correlation between HE‐related keywords in the race and ethnicity category and the US keyword. The prominent role of developed countries in these publications may result from their enhanced economic power. Despite the marked increase in research publications in HE internationally, there exists an intensifying disparity in research activities that is closely linked to the income categories of various countries. Furthermore, the difference can be attributed to the access that researchers from developed countries have to publishers' networks and the official support they receive in this regard [23]. This discrepancy can also be partially attributed to the historically high expenses of publishing and subscribing to top‐tier international journals, which may have impeded certain low‐income countries from sharing their research findings [24, 25].

This study revealed the prominent knowledge areas in HE, as recognized through co‐occurrence network analysis and the delineation of topic clusters. The five thematic clusters associated with the period preceding the COVID‐19 pandemic are “Health care,” “Health economics,” “Race and ethnicity,” “Social determinants of health,” and “Age and gender.” These titles and their associated topics reveal that a considerable amount of research in HE before 2020 has been dedicated to exploring the fundamental determinants of equity and inequity in health. During the early 1990s, the progression of research in HE fostered a deeper insight into equality. Accordingly, researchers analyzed HE issues from a global and national perspective, leveraging the framework of health systems theory [23]. This is even though healthcare systems represent only one of several intermediary factors, and HE status may also be influenced by various other determinants. The WHO asserts that the SDH are the most significant contributors to achieving equitable health outcomes, as they directly influence individuals' access to resources and their power dynamics [26]. Throughout the past few years, WHO has been promoting the concept that member countries should generate knowledge, gather and analyze evidence, and conduct research on policies and practical approaches to mitigate disparities in SDHs and health inequities [27]. Consequently, the worldwide consensus on evaluating SDHs and investigating the effects of socioeconomic factors on health disparities has prompted rapid growth in studies related to HE [28].

The analysis of the co‐occurrence network concerning research keywords in HE from 2020 to 2023, following the onset of COVID‐19 pandemic, resulted in the identification of six main topic clusters, namely “Health workforce,” “Risk factors,” “Maternal and child health,” “COVID‐19,” “Cancer,” and “Mental health.” The health workforce has been recognized as the leading topic cluster following 2020. It is vital in facilitating access to healthcare services and profoundly affects the fairness of service delivery and overall health outcomes. The shortage of healthcare personnel has continually been a barrier for countries in their efforts to fulfill the Millennium Development Goals [29, 30]. The results suggest that the predominant focus of HE research over the past 5 years has been on the burden of different diseases and their associated risk factors. The role of disease burden is pivotal in the analysis of HE, underscoring the importance of addressing disparities to attain fair and just health outcomes for all communities [31]. Vulnerable populations are particularly susceptible to the exacerbating effects of disease burden, which can lead to increased health inequities [32]. Evidence suggests that health inequities are present at the international and national levels, marked by substantial disparities in the disease burden and life expectancy. Understanding these disparities is crucial for addressing HE and guiding targeted interventions for improved health outcomes [33]. Moreover, promoting HE presents significant challenges due to the escalating disparities in exposure to various health risk factors [6]. Although substantial advancements have been made in recognizing health inequities, continuous efforts must be undertaken to ensure that healthcare systems effectively address the root causes of these disparities.

Based on the frequency of keywords, the study's findings highlighted that the four key emerging topics in HE before 2020 included “Disease burden,” “Telemedicine,” “Sex factor,” and “Sexual and gender minorities.” The emergence of the disease burden as a prominent issue in the years before 2020, when analyzed in conjunction with findings from the topic clusters of the previous 5 years, suggests a notable trend in HE research toward various diseases. In parallel, across many countries, particularly those with low income, there is still a pressing need for evidence that addresses the systemic factors and socioeconomic determinants influencing health disparities. Although telemedicine and e‐health have great potential to promote health outcomes and HE [34, 35], it might contribute to increased disparities, primarily due to challenges related to digital literacy and technology accessibility, which mainly influence older patients and those from low‐income backgrounds [36]. Research indicates that individuals with varying gender identities have experienced increased mental and physical health issues [37]. Future studies in HE that address sexual and gender minority groups should concentrate on the creation of new frameworks for evaluating discrimination across individual, interpersonal, and structural levels. These research efforts should aim to establish evidence‐based interventions that can reduce discrimination and its associated adverse effects [38].

This study showed that “Artificial intelligence,” “Racial disparity,” “Machine learning,” and “COVID‐19,” were four leading emerging topics of HE pertaining to the period following the onset of the COVID‐19 pandemic. Artificial intelligence (AI) has made its mark on health systems, paralleling the effects observed in numerous other fields [39]. Integrating AI in healthcare offers significant opportunities and challenges concerning HE [40]. While AI can result in enhanced patient outcomes and assist in the efficiency of professional duties [40], it could deepen existing health disparities. Significant equity considerations include the features of the data, the design of the models, their implementation, and the relevant contextual background [41]. Stakeholders should place a high priority on equity during the development and application of AI in the healthcare sector to optimize its ability to improve health outcomes for all individuals. The issue of structural racism has significantly affected HE, resulting in ongoing health inequalities and negative health results for marginalized groups. Combating structural racism is essential to promoting equality and fair opportunities for a healthy life for all [42]. Moreover, healthcare systems are required to address the impact of racism in sustaining disparities across education, housing, employment, and the judicial systems [43]. The emergence of the COVID‐19 pandemic has intensified worries about the escalating health inequities associated with the disease. Concerning this issue, more than 2000 articles have been published in fewer than 22 months, specifically between January 2020 and October 2021 [13]. The health inequities highlighted by the COVID‐19 pandemic originate from differences in exposure, transmission, susceptibility, and treatment, which are intrinsically linked to broader structural and systemic inequities [44]. As the frequency of emerging infectious diseases continues to rise, it is crucial to analyze the relationship between socioeconomic inequalities and pandemics, as this understanding is fundamental for future preparedness and alleviating health disparities [45].

## 5 Limitations of the Study

This bibliometric research focused exclusively on data from four leading databases, without incorporating local databases. As a result, this issue might have resulted in the exclusion of specific articles from this study.

## 6 Conclusions

There has been a significant increase in research related to HE in recent years, with a considerable share of these studies being carried out in high‐income countries. The evidence reveals that the focus of research in HE has shifted from the fundamental aspects of equity in health, such as service delivery system and SDH, to an emphasis on the impact of various diseases and the risk factors contributing to these conditions. Moreover, AI, racial disparity, and machine learning are three emerging topics in HE in the last 5 years. Emerging topics represent significant areas of interest as novel research domains, particularly within low‐ and middle‐income countries.

## Author Contributions

**Yaser Sarikhani:** conceptualization, formal analysis, supervision, writing – review and editing, validation. **Arefeh Kalavani:** methodology, software, funding acquisition, formal analysis. **Seyede Maryam Najibi:** writing – original draft, writing – review and editing, conceptualization, validation. All authors have read and approved the final version of the manuscript.

## Ethics Statement

This study is approved by the Ethics Committee of Jahrom University of Medical Sciences under the code IR.JUMS.REC.1401.051.

## Conflicts of Interest

The authors declare no conflicts of interest.

## Transparency Statement

The lead author Yaser Sarikhan affirms that this manuscript is an honest, accurate, and transparent account of the study being reported; that no important aspects of the study have been omitted; and that any discrepancies from the study as planned (and, if relevant, registered) have been explained.

## Data Availability

The data for the current research were extracted from the Scopus, PubMed, ScienceDirect, and Web of Science databases and subsequently saved in RIS format files. The corresponding author will provide these files to the journal upon request. Seyede Maryam Najibi had full access to all of the data in this study and took complete responsibility for the integrity of the data and the accuracy of the data analysis.
